# Processes, contexts, and rationale for disinvestment: a protocol for a critical interpretive synthesis

**DOI:** 10.1186/2046-4053-3-143

**Published:** 2014-12-11

**Authors:** Michael G Wilson, Moriah E Ellen, John N Lavis, Jeremy M Grimshaw, Kaelan A Moat, Joshua Shemer, Terry Sullivan, Sarah Garner, Ron Goeree, Roberto Grilli, Justin Peffer, Kevin Samra

**Affiliations:** McMaster Health Forum, McMaster University, Hamilton, Canada; Centre for Health Economics and Policy Analysis, McMaster University, Hamilton, Canada; Department of Clinical Epidemiology and Biostatistics, McMaster University, Hamilton, Canada; Jerusalem College of Technology, Jerusalem, Israel; Gertner Institute for Epidemiology and Health Policy Research, Israeli Center for Technology Assessment in Health Care, Tel Hashomer, Israel; Department of Political Science, McMaster University, Hamilton, Canada; Department of Global Health and Population, Harvard School of Public Health, Cambridge, USA; Clinical Epidemiology Program, Ottawa Hospital Research Institute, Ottawa, Canada; Department of Medicine, University of Ottawa, Ottawa, Canada; Institute of Population Health, University of Ottawa, Ottawa, Canada; Sackler School of Medicine, Tel Aviv University, Tel Aviv, Israel; Institute for Health Policy Management & Evaluation, University of Toronto, Toronto, Canada; National Institute for Health and Care Excellence, London, UK; Program for Assessment of Technology in Health, McMaster University, Hamilton, Canada; Emilia-Romagna Regional Agency for Health and Social Care, Bologna, Italy; Ontario Ministry of Health and Long-Term Care, Toronto, Canada; British Columbia Ministry of Health, Victoria, Canada

**Keywords:** Disinvestment, Critical interpretive synthesis, Health technologies, Health system context, Policy development, Policy implementation, Reassessment, Appropriateness

## Abstract

**Background:**

Practical solutions are needed to support the appropriate use of available health system resources as countries are continually pressured to ‘do more with less’ in health care. Increasingly, health systems and organizations are exploring the reassessment of possibly obsolete, inefficient, or ineffective health system resources and potentially redirecting funds to those that are more effective and efficient. Such processes are often referred to as ‘disinvestment’. Our objective is to gain further understanding about: 1) whether how and under what conditions health systems decide to pursue disinvestment; 2) how health systems have chosen to undertake disinvestment; and 3) how health systems have implemented their disinvestment approach.

**Methods/Design:**

We will use a critical interpretive synthesis (CIS) approach, to develop a theoretical framework based on insights drawn from a range of relevant sources. We will conduct systematic searches of databases as well as purposive searches to identify literature to fill conceptual gaps that may emerge during our inductive process of synthesis and analysis. Two independent reviewers will assess search results for relevance and conceptually map included references. We will include all empirical and non-empirical articles that focus on disinvestment at a system level. We will then extract key findings from a purposive sample of articles using frameworks related to government agendas, policy development and implementation, and health system contextual factors and then synthesize and integrate the findings to develop a framework about our core areas of interest. Lastly, we will convene a stakeholder dialogue with Canadian and international policymakers and other stakeholders to solicit targeted feedback about the framework (e.g., by identifying any gaps in the literature that we may want to revisit before finalizing it) and deliberating about barriers for developing and implementing approaches to disinvestment, strategies to address these barriers and about next steps that could be taken by different constituencies.

**Discussion:**

Disinvestment is an emerging field and there is a need for evidence to inform the prioritization, development, and implementation of strategies in different contexts. Our CIS and the framework developed through it will support the actions of those involved in the prioritization, development, and implementation of disinvestment initiatives.

**Systematic review registration:**

PROSPERO CRD42014013204

**Electronic supplementary material:**

The online version of this article (doi:10.1186/2046-4053-3-143) contains supplementary material, which is available to authorized users.

## Background

Practical solutions are needed to support the appropriate use of available health system resources as countries are continually pressured to ‘do more with less’ in health care. Countries are facing the challenge of how to maintain high quality care in the face of shrinking or slow-growing budgets [1, 2]. Due to limited resources, health systems and organizations are exploring the reassessment of possibly obsolete, inefficient, or ineffective health technologies (e.g., drugs, procedures, and devices) and potentially redirecting funds to more effective and efficient health technologies [3]. Such processes are often referred to as ‘disinvestment’, which is defined as ‘…the processes of (partially or completely) withdrawing health resources from any existing health care practices, procedures, technologies or pharmaceuticals that are deemed to deliver little or no health gain for their cost, and thus are not efficient health resource allocations’ [4, 5].

Significant effort has been invested in developing well-defined criteria and processes that draw on the best available evidence to assess the safety, effectiveness, and cost-effectiveness of new and emerging health technologies [6]. Yet similar efforts have not been directed towards disinvestment of 1) technologies that are believed to be ineffective or inefficient as compared to other technologies; 2) technologies that have never been adequately assessed and may not be beneficial but have already been incorporated into health care delivery; and 3) technologies that were initially worthy of investment but that have become obsolete. Failure to engage in disinvestment leads to inefficient allocation of limited health resources because health systems continue to fund technologies (and remunerate those who deliver them) that may provide limited or no health gain.

No widely accepted disinvestment method has been developed [7–10], but some HTA agencies have started to develop disinvestment lists focused on ineffective, harmful, or non-cost-effective health care practices [11–13] and criteria have been proposed for prioritizing health technologies for reassessment [11, 14]. Proposed methods for disinvestment include adopting a health technology reassessment approach [15], using Cochrane reviews as a guide [16, 17] or using frameworks such as the Guideline for Not Funding Health Technologies and the Program Budgeting and Marginal Analysis method. In Canada (where this synthesis has been funded), there have been initiatives aimed at prioritizing, developing, and implementing approaches to disinvestment both nationally (e.g., identifying obsolete technologies) [3, 6] and provincially, including efforts in Ontario to apply the Choosing Wisely Campaign from the United States [18] and to develop a reassessment framework [19, 20]; in British Columbia where 44 disinvestment initiatives were implemented [21]; and in Alberta where $40 million in savings was realized due to efficiency gains [22].

Notwithstanding these examples, disinvestment is an emerging field and there is a paucity of evidence to inform the prioritization, development, and implementation of strategies in different contexts. For example, two existing reviews [3, 9] do not focus on rationale-, context-, and process-related factors that need to be taken into consideration by policymakers and stakeholders involved in efforts to prioritize, develop, and implement disinvestment approaches. Considering the lack of theoretical development [3], our CIS will support the actions of those involved in the prioritization, development, and implementation of disinvestment initiatives.

### Objectives

The goal of this knowledge synthesis is to conduct a critical interpretive synthesis (CIS) to gain further understanding about:

1. whether, how, and under what conditions health systems decide to pursue disinvestment (i.e., agenda setting or prioritization);
2. how health systems have chosen to undertake disinvestment (i.e., policy development); and
3. how health systems have implemented their disinvestment approach (i.e., policy implementation).

Our overarching objective is to develop an explanatory framework about disinvestment related to each of the three principal aims to contribute to efforts for tailoring future disinvestment initiatives in different countries so that appropriate initiatives can be matched to context.

## Methods/Design

We will use the CIS approach, which (in general) has the core objective of developing a theoretical framework based on insights and interpretation drawn from a broad range of relevant sources (i.e., not just those that meet particular design or quality criteria). CIS is ideal when the question addressed is likely to draw on literature that is not well developed or focused [23, 24], which is the case with the literature related to the rationale, context, and processes for the prioritization, policy development, and implementation of disinvestment. Table 1 compares the key features of the CIS approach to those of traditional systematic reviews.

**Table 1:** **Comparison of key features of traditional systematic reviews and CIS**

Synthesis feature	Traditional systematic review	Critical interpretive synthesis
Research question	• A research question is developed a priori and is not changed during the course of the research study.	• A compass question is developed, which can evolve over the course of the research study, in a transparent manner.
Literature search	• Exhaustive literature search	• Exhaustive literature search but can purposively sample literature outside of original search parameters
Article selection	• Static inclusion and exclusion criteria are applied to search results;	• Inclusion and exclusion criteria are iteratively refined to reflect the evolving compass questions (often through successive pilot testing of the criteria using small batches of references with the final criteria applied to all search results);
	• Included articles are empirical studies and often consist only of a small number of specific methodological designs (e.g., RCT, CCT, observational studies); and	• Included articles can consist of both empirical and non-empirical (e.g., editorials, essays) analyses that offer insight into the topic under investigation (i.e., articles are not limited to those that meet certain design or quality criteria); and
	• All relevant articles meeting inclusion criteria and minimum quality standard are included in the analysis.	• A purposive sample of eligible articles is typically selected based on the unique insights they can provide to the topic under investigation.
Quality rating of articles	• Quality ratings are usually applied to all articles included in a systematic review and used to help interpret the findings.	• Relevance ratings are applied to articles, not quality ratings (i.e., articles are rated on their ability to answer the compass question).
Synthesis	• Data is extracted from all studies meeting the inclusion criteria using an a priori approach; and	• Data is extracted from a purposive sample of studies using an analytic framework where the constructs are iteratively revised and reapplied to the included papers to ensure the constructs are grounded in the data; and
	• Findings are synthesized either quantitatively (i.e., in a meta-analysis) or qualitatively, which typically involves reporting findings from studies according to the pre-defined outcomes of interest.	• The analytical constructs that emerged from the iterative analysis are used to derive an explanatory framework.

We have adopted an approach that complements the methods used by traditional systematic reviews with purposive sampling and inductive analysis, which are key features of CIS methods. In general, this will include:

1. conducting systematic searches of the literature (later complemented by purposive searches to fill conceptual gaps):
2. reviewing articles for inclusion;
3. purposively sampling articles based on their ability to inform key conceptual areas related to disinvestment;
4. extracting key findings according to frameworks related to government agendas, policy development and implementation and health system contextual factors; and
5. synthesizing and integrating findings to develop a framework about our core areas of interest.

As a result, we will employ both a very explicit and structured approach to search and review the indexed literature (similar to traditional systematic reviews) while also borrowing the inductive methods often associated with qualitative research designs, to ensure that our final sample of included papers and the framework derived from them is theoretically rich and relevant to our objectives.

### Literature search

We will search 15 databases that index research literature on a diversity of subject domains to ensure we identify articles addressing a broad spectrum of factors related to rationale, context, and processes for disinvestment prioritization, policy development, and implementation. The 15 databases include: MEDLINE (1946–present), Embase (1974–2014 April 29), Healthstar (1966 to March 2014), International Political Science Abstracts (1989–present), PsycINFO (1987 to April Week 4 2014), The Cochrane Library (Issue 4 of 12, April 2014 and Issue 1 of 4, Jan 2014), CINAHL (1982–present), Social Science Abstracts, Applied Social Sciences Index and Abstracts (1987–present), PAIS International (1914–present), ProQuest Political Science (1985–present), Sociological Abstracts (1952–present), Worldwide Political Science Abstracts (1975–present), Web of Science Core Collection (1976–present), and PubMed (for non-MEDLINE records). We have provided our detailed search strategy in Additional file 1, which was developed in consultation with a library scientist at the Program for Assessment of Technology in Health (PATH) at McMaster University and then peer reviewed before being finalized. In addition, we will contact experts in the field (including our project team of researchers and knowledge users), search websites of organizations associated with HTAi and INAHTA, and search the reference lists of acquired publications to identify additional published and unpublished literature.

We will also conduct purposive searches to identify literature to fill conceptual gaps that may emerge during our inductive process of synthesis and analysis. We anticipate conducting additional purposive searches after we have conceptually mapped the relevant articles included in our sample frame in the article selection stage and again during our inductive constant comparative approach to analysis of the included papers. These searches will be developed collaboratively with the project team and will be based on our increased familiarity with the literature and collective expertise.

### Selection criteria development

We will include all empirical and non-empirical articles that focus on disinvestment at a system level. This includes macro (i.e., national and sub-national) and meso (i.e., regions, health care organizations, or networks) levels but not micro level (i.e., individual clinicians or teams of clinicians).

### Reference reviewing and article selection

#### Step 1 - Reviewing

We will review the titles and abstracts of all references captured by our search strategy. Each reference will be assessed in duplicate by two of us (MGW and MEE). The references will first be classified as ‘potentially relevant’ or ‘exclude’. We will then retrieve the full-text of all non-excluded articles and review them independently in duplicate to make a final assessment for whether they are relevant to disinvestment based on the broad areas outlined in our principal aims. The papers deemed to be relevant will be included in our sample frame of literature from which we will draw our purposive sample for the synthesis.

#### Step 2 - Conceptual mapping

We will conceptually map the relevant papers using a standardized form (see Additional file 2) based on our data extraction categories outlined below and in more detail in Additional file 3. Specifically, we will categorize the papers using a 3 × 3 matrix that cross links rationale, political, and health system contexts and process against our principle areas of interest (prioritization, policy development, and implementation). This mapping exercise will be similar to those often used in scoping reviews whereby the literature is categorized into domains and topics of interest to facilitate assessments of the state the literature (for an example, see Wilson et al.) [25].

Each paper will be assessed using the standardized form by one reviewer, which will be independently checked by one of us (MGW or MEE) who will, in addition to the categories included in the conceptual mapping, review each paper and identify those that are the most relevant and likely to offer important conceptual insights that will help answer our research questions. We will use the following broad question to guide this assessment: does the paper provide clear insights into rationale, contexts, and/or processes related to why health systems pursue disinvestment and how they engage in and implement approaches to disinvestment?

#### Step 3 - Purposive sampling

We will use this mapping exercise to identify areas that are conceptually rich and areas where there appear to be conceptual gaps, which we will then use to guide our selection of a purposive sample of relevant papers. Specifically, project leads (MGW, MEE, JS, and TS) will review the results of the conceptual mapping and the assessments of which papers are likely to offer important conceptual insights and select a proposed set of papers to be included in the analysis, which we will then seek feedback about from the full project team. In addition, the project leads will develop a draft plan for further purposive searches (as outlined below) to help fill areas where there are conceptual gaps. We will then share the sample selection and the draft plan for further purposive searches with the full project team and convene a meeting to collectively review and make adjustments to the sample and search approach. Lastly, additional stages of purposive sampling may be conducted during the synthesis and integration of findings phase as conceptual gaps in the analysis are identified.

### Data extraction

We will extract information from each article included in our synthesis by writing a 1–2 paragraph summary of key messages about each and then extracting specific findings according to frameworks of government agenda setting (as a set of variables that can help explain why health systems pursue disinvestment), policy development and implementation (as a set of variables that can help explain how health systems develop and implement approaches to disinvestment), and health systems (as a set of cross-cutting variables about rationale, context, and processes). We have drawn the government agenda setting factors from Kingdon’s widely used and empirically tested model that identifies three ‘streams’ of factors: 1) factors contributing to framing the problem as something deserving government attention; 2) availability of viable policies or solutions to address the problem; and 3) whether the prevailing ‘politics’ garner government attention [26]. For policy development and implementation factors, we will use the ‘3I’ framework to extract and categorize relevant information. The 3I framework is derived from the political science literature which broadly relates to how political institutions (e.g., government decision-making structures and processes), interests (i.e., groups with a vested interest), and ideas (i.e., values and research-based knowledge) affect the actions of those making political decisions [27]. For health system factors, we will use a framework of governance, financial, and delivery arrangements within health systems that we have used to categorize systematic reviews in Health Systems Evidence ( http://www.healthsystemsevidence.org).

We will also extract the characteristics of each article, including the publication date, study time period (if applicable), type of paper (e.g., primary research versus non-primary research such as editorials), methods used (if applicable), country focus (if applicable), and the academic discipline (e.g., health services, systems and policy, economics, health technology assessment). We have outlined the detailed questions for the study characteristics and for each of the frameworks that we will use during data extraction in Additional file 3.

### Synthesizing and integrating findings

Based on qualitative research methods, we will use a constant comparative method throughout our analysis to develop an explanatory framework of disinvestment, which will allow us to ensure our framework is grounded in the data from the included papers but still drawing on the collective interdisciplinary expertise and experience of our study team. This will involve:

1. identifying common themes and concepts based on our summaries of and data extracted from each paper;
2. developing theoretical constructs based on the emerging themes and concepts;
3. critiquing the emerging theoretical constructs as a whole and with our full sample of literature to identify conceptual gaps in the available evidence in relation to our principal aims;
4. conducting additional purposive sampling of included papers and/or conducting additional purposive searches to fill conceptual gaps (if needed) until theoretical saturation is reached [28]; and
5. integrating the theoretical constructs into a ‘synthesizing argument’ about disinvestment (i.e., an explanatory framework).

Each of these stages is iterative in nature and the process will involve ongoing consultation with members of our team.

## Discussion

### Challenges

We anticipate that our biggest challenge will be to organize and synthesize findings across a diverse set of papers. Our mitigation strategy for this challenge has been to adopt a methodology that is designed to synthesize findings in complex topic areas and to assemble an interdisciplinary team that is keen to collaborate and can provide a broad range of methodological, analytical, and policy expertise. In addition, our plan for a full-day interdisciplinary workshop (described in our KT section) will engage policymakers and other stakeholders involved with supporting processes for and decisions about disinvestment. We anticipate that the workshop will be important for addressing any remaining challenges related to the analysis, framework development, and interpretation.

### Knowledge translation

Our knowledge translation (KT) plan emphasizes our commitment to stakeholder engagement (i.e., integrated KT) as well as using a multi-faceted approach to mobilizing the findings to ensure knowledge users can efficiently find and use relevant and high-quality research evidence when needed.

As part of our commitment to integrated KT, we have assembled an interdisciplinary team of researchers and knowledge users who will collaboratively work together to ensure the project is timely and relevant, as well as progressing as intended and collectively address any methodological, analytical, and practical issues that arise. The project’s research leads (MGW and ME) will engage the full team through teleconferences and in-person at meetings where most of us will already be present (e.g., the Canadian Association of Health Services and Policy Research Annual Conference) to provide input on the inclusion criteria, results of the conceptual mapping, purposive sampling and searching, synthesis and analysis, framework, final paper, and format and content for the stakeholder dialogue (outlined in more detail below).

By drawing on the diverse expertise of our team members, we will be able to engage in interdisciplinary collective problem-solving that will ensure the review provides relevant and user-friendly results that can be used to inform policy and practice. Through engagement and collaboration with our interdisciplinary team of knowledge users and researchers in the development of this proposal, we have already begun integrated KT by ensuring the types of information sought, analyzed, and disseminated will be relevant to informing disinvestment processes.

The centerpiece of our end-of-grant KT activities will be to convene a 1-day stakeholder dialogue at the McMaster Health Forum, which will be facilitated by one of us (Lavis). We will engage approximately 18–22 participants in the stakeholder dialogue which, based on our experience with running more than 30 stakeholder dialogues, is optimal for ensuring the deliberations include a breadth of perspective but also allow for meaningful contributions from all participants. We will aim to engage Canadian and international policymakers and other stakeholders involved with supporting processes for and decisions about disinvestment. In addition, we will engage researchers from a range of disciplines (e.g., public policy, health economics, health technology assessment, knowledge translation) involved with disinvestment. Prior to the dialogue, we will produce a draft report of the synthesis, which we will distribute to participants approximately 2 weeks before convening. During the dialogue we will:

1. solicit feedback and deliberate about the key elements of the framework derived from the synthesis;
2. identify and deliberate about barriers for disinvestment processes and strategies to realistically address these barriers; and
3. identify and deliberate about next steps that could be taken by different constituencies to move forward with disinvestment.

Following the dialogue, we will review the suggestions from participants and make changes to the framework only after revisiting the literature to ensure its elements remain grounded in the available evidence. We will disseminate the final report through the networks of each of our team members and produce and submit at least one manuscript to a peer-reviewed journal (open access).

## Electronic supplementary material

Additional file 1: **Literature search strategy.** Search strategy used to identify literature for the critical interpretive synthesis. (DOCX 23 KB)

Additional file 2: **Conceptual mapping form for article selection process.** Questions and categories used to conduct the conceptual mapping phase of the critical interpretive synthesis. (DOCX 15 KB)

Additional file 3: **Data extraction framework.** Approached to be used to extract data from included literature. (DOCX 26 KB)

## Abbreviations

HTA: Health technology assessment
CIS: Critical interpretive synthesis
HTAi: Health Technology Assessment International
INAHTA: International Network of Agencies for Health Technology Assessment
KT: Knowledge translation.
