# Supplementary material for: Processes, contexts, and rationale for disinvestment: a protocol for a critical interpretive synthesis
Source: Syst Rev. 2014 Dec 11;3:143. doi: 10.1186/2046-4053-3-143 (PMC4273322; doi:10.1186/2046-4053-3-143)
Supplement: Supplementary file 1 — Additional file 1: Literature search strategy. Search strategy used to identify literature for the critical interpretive synthesis. (DOCX 23 KB) [file 13643_2014_309_MOESM1_ESM.docx]

**Additional File 1: Literature Search Strategy**

The literature search was performed by an information specialist using a peer-reviewed search strategy.

Published literature was identified by searching the following bibliographic databases: MEDLINE (1946-present) with in-process records, Embase (1974-2014 April 29), Healthstar (1966 to March 2014), International Political Science Abstracts (1989-present), and PsycINFO (1987 to April Week 4 2014) via Ovid; The Cochrane Library (Issue 4 of 12, April 2014 & Issue 1 of 4, Jan 2014) via Wiley; CINAHL (1982-present) and Social Science Abstracts via EBSCO; Applied Social Sciences Index and Abstracts (1987-present), PAIS International‎ (1914-present), ProQuest Political Science‎ (1985-present), Sociological Abstracts‎ (1952-present), and Worldwide Political Science Abstracts‎ (1975-present) via ProQuest; Web of Science Core Collection (1976-present) from Thomson Reuters; and, PubMed (for non-MEDLINE records).  The search strategy was comprised of both controlled vocabulary, such as the National Library of Medicine’s MeSH (Medical Subject Headings), and keywords. The main search concepts were disinvestment and its synonyms, combined with contextual use terms such as economic evaluation, resource allocation, health, healthcare, etc.

No methodological filters or limits were applied. See Appendix 1 for the detailed search strategies.

The initial search was completed on April 30, 2014. A search update will be performed on July 31, 2014.

| **OVERVIEW** | |  | |
| --- | --- | --- | --- |
| Interface: | | Ovid | |
| Databases: | | Healthstar <1966 to March 2014> (hstr)  MEDLINE In-Process & Other Non-Indexed Citations and Ovid MEDLINE <1946 to Present> (prmz)  PsycINFO <1987 to April Week 4 2014> (psyc3,psyc4,psyc5,psyc6,psyc7,psyc8)  Embase <1974 to 2014 April 29> (oemezd) | |
| Date of Search: | | April 30, 2014 | |
| Alerts: | | Monthly search updates began April 30, 2014 and ran until July 31, 2014 | |
| Study Types: | | All | |
| Limits: | | None | |
| **SYNTAX GUIDE** | | |  |
| / | At the end of a phrase, searches the phrase as a subject heading | | |
| exp | Explode a subject heading | | |
| * | Before a word, indicates that the marked subject heading is a primary topic;  or, after a word, a truncation symbol (wildcard) to retrieve plurals or varying endings | | |
| # | Truncation symbol for one character | | |
| ? | Truncation symbol for one or no characters only | | |
| ADJ | Requires words are adjacent to each other (in any order) | | |
| ADJ# | Adjacency within # number of words (in any order) | | |
| .ti | Title | | |
| .ab | Abstract | | |
| .mp | Title, Abstract, Subject Heading, Heading Word, Trade Name, Other Title, Device Manufacturer, Drug Manufacturer, Device Trade Name, Keyword, Name of Substance Word, Keyword Heading Word | | |

| **MULTI-DATABASE (OVID) SEARCH STRATEGY** |
| --- |

| **Line #** | **Search Terms** | **Results** |
| --- | --- | --- |
| 1 | (disinvest* or dis-invest*).mp. | 447 |
| 2 | ((Program* adj Budgeting adj Marginal Analys#s) or PBMA).mp. | 358 |
| 3 | (Health Technology Reassessment* or Health Technology Re-assessment*).ti,ab. | 15 |
| 4 | or/1-3 | 785 |
| 5 | ((cost-ineffective* or costineffective* or decommission* or de-commission* or delist* or de-list* or (decremental* adj cost adj effectiv*) or exnovation* or ex-novation* or ineffective* or in-effective* or (little adj2 value) or "low-value" or (marginal* adj2 (valu* or (cost adj effective*) or analys*)) or "no longer effective" or obsolete* or obsolescen* or outdated or out-dated or outmoded or out-moded or out-of-date* or (reduc* adj2 (coverage* or use*)) or resource release or suboptimal* or sub-optimal* or (substitut* adj technolog*) or supersed* or superced*) adj5 (activit* or biotechnolog* or bio-technolog* or care or device* or drug or drugs or healthcare or health-care or intervention* or medical or medicine* or nondrug* or non-drug* or pharmaceutical* or procedure* or practice* or practis* or service* or technolog* or test* or therap* or treatment*)).ti. | 4202 |
| 6 | ((cost-ineffective* or costineffective* or decommission* or de-commission* or delist* or de-list* or (decremental* adj cost adj effectiv*) or exnovation* or ex-novation* or ineffective* or in-effective* or (little adj2 value) or "low-value" or (marginal* adj2 (valu* or (cost adj effective*) or analys*)) or "no longer effective" or obsolete* or obsolescen* or outdated or out-dated or outmoded or out-moded or out-of-date* or (reduc* adj2 (coverage* or use*)) or resource release or suboptimal* or sub-optimal* or (substitut* adj technolog*) or supersed* or superced*) adj2 (activit* or biotechnolog* or bio-technolog* or care or device* or drug or drugs or healthcare or health-care or intervention* or medical or medicine* or nondrug* or non-drug* or pharmaceutical* or procedure* or practice* or practis* or service* or technolog* or test* or therap* or treatment*)).ab. | 30807 |
| 7 | or/5-6 | 34584 |
| 8 | Comparative Effectiveness Research/ use prmz,hstr | 2958 |
| 9 | Comparative Effectiveness/ use oemezd | 9773 |
| 10 | "Costs and Cost Analysis"/ use prmz,hstr | 81954 |
| 11 | Economic Evaluation/ use oemezd | 9137 |
| 12 | Health Care Economics/ use psyc3,psyc4,psyc5,psyc6,psyc7,psyc8 | 415 |
| 13 | exp "Costs and Cost Analysis"/ use psyc3,psyc4,psyc5,psyc6,psyc7,psyc8 | 17229 |
| 14 | Cost-Benefit Analysis/ use prmz,hstr | 118157 |
| 15 | "Cost Benefit Analysis"/ use oemezd | 64019 |
| 16 | "Cost Effectiveness Analysis"/ use oemezd | 96664 |
| 17 | Decision-Making/ use prmz,hstr | 136677 |
| 18 | Decision Making/ use oemezd | 138297 |
| 19 | exp Decision Making/ use psyc3,psyc4,psyc5,psyc6,psyc7,psyc8 | 55733 |
| 20 | Decision Making, Organizational/ use prmz,hstr | 20816 |
| 21 | *Organization/ use oemezd | 31629 |
| 22 | Decision Support Techniques/ use prmz,hstr | 23792 |
| 23 | *Decision Support System/ use oemezd | 6588 |
| 24 | Financial Management/ use prmz,hstr | 30608 |
| 25 | Financial Management/ use oemezd | 100006 |
| 26 | Health Expenditures/ use prmz,hstr | 27109 |
| 27 | exp *"Health Care Cost"/ use oemezd | 47418 |
| 28 | Health Planning/ use prmz,hstr | 40787 |
| 29 | Health Priorities/ use prmz,hstr | 17480 |
| 30 | Health Resources/ use prmz,hstr | 17463 |
| 31 | Health Care Planning/ use oemezd | 77612 |
| 32 | exp Resource Allocation/ use prmz,hstr | 30625 |
| 33 | Resource Allocation/ use oemezd | 15160 |
| 34 | Resource Management/ use oemezd | 7522 |
| 35 | Resource Allocation/ use psyc3,psyc4,psyc5,psyc6,psyc7,psyc8 | 2228 |
| 36 | exp "Cost Control"/ use prmz,hstr | 57024 |
| 37 | *"Cost Control"/ use oemezd | 6381 |
| 38 | Cost Containment/ use psyc3,psyc4,psyc5,psyc6,psyc7,psyc8 | 462 |
| 39 | exp Technology Assessment, Biomedical/ use prmz,hstr | 18272 |
| 40 | Biomedical Technology Assessment/ use oemezd | 11348 |
| 41 | (allocat* or ration or rationing).ti,ab. | 238229 |
| 42 | (budget* or cost* or expenditure* or expens* or pharmacoeconomic* or (pharmaco adj economic*) or pric* or reimburs* or value).ti. | 608424 |
| 43 | "Comparative Effectiveness Research".ti,ab. | 3380 |
| 44 | ((cost or costs) adj1 (benefit* or beneficial or effective*)).ti,ab. | 273054 |
| 45 | (decision* adj1 (make or makes or making or support or supports or supporting or inform or informs or informed or informing)).ti,ab. | 303495 |
| 46 | economic*2 evaluation*.ti,ab. | 22372 |
| 47 | ((expenditure? or funding or funds or resource?) adj2 (reallocat* or re-allocat* or redeploy* or re-deploy*)).ti,ab. | 1111 |
| 48 | (HTA or HTAs or technolog*).ti,ab. | 820005 |
| 49 | (priority or priorities or prioritiz* or prioritis*).ti,ab. | 208214 |
| 50 | or/8-49 | 2988545 |
| 51 | (biomedical* or bio-medical* or health*).mp. | 8313636 |
| 52 | care.ti,ab. | 2886363 |
| 53 | or/51-52 | 9495616 |
| 54 | (4 and (50 or 53)) or (7 and 50 and 53) | 3476 |
| 55 | remove duplicates from 54 | 1738 |

| **OTHER DATABASES** | | |
| --- | --- | --- |
| International Political Science Abstracts (Ovid) | Same keywords used as per Medline search. |  |
| Cochrane Library, including: CDSR, Methods Register, HTA Database & NHSEED  Issue 4, April 2014 & 1 of 4, Jan 2014 (Wiley) | Same MeSH and keywords used as per Medline search. Syntax adjusted for Cochrane Library databases. |  |
| CINAHL & Social Science Abstracts (EBSCO) | Same MeSH and keywords used as per Medline search. Syntax adjusted for EBSCO interface. |  |
| Sociological Abstracts, Applied Social Sciences Index and Abstracts, Worldwide Political Science Abstracts, PAIS, Political Science, A&I Theses (ProQuest) | Same keywords used as per Medline search. Syntax adjusted for Web of Science. |  |
| Web of Science Core Collection  (Thomson Reuters) | Same keywords used as per Medline search. Syntax adjusted for Web of Science. |  |
| PubMed, for non-Medline records  (NLM) | Same MeSH and keywords used as per Medline search. Syntax adjusted for National Library of Medicine interface. |  |

| **GREY LITERATURE** | |
| --- | --- |
| Dates for Search: | April 30, 2014 |
| Keywords: | Included terms for disinvestment and its synonyms |
| Limits: | None |

Library & Archives Canada, Theses Canada: <http://amicus.collectionscanada.gc.ca/thesescanada-bin/Main/BasicSearch?coll=18&l=0&v=1> (grey lit. or database)

OAIster (grey literature): <http://oaister.worldcat.org/advancedsearch>
