# Supplementary material for: Processes, contexts, and rationale for disinvestment: a protocol for a critical interpretive synthesis
Source: Syst Rev. 2014 Dec 11;3:143. doi: 10.1186/2046-4053-3-143 (PMC4273322; doi:10.1186/2046-4053-3-143)
Supplement: Supplementary file 2 — Additional file 2: Conceptual mapping form for article selection process. Questions and categories used to conduct the conceptual mapping phase of the critical interpretive synthesis. (DOCX 15 KB) [file 13643_2014_309_MOESM2_ESM.docx]

**Additional File 2 – Conceptual Mapping Form for Article Selection Process**

Completed by: _____________________

1. **Global assessment question** (to be completed by PIs): Does the paper provide clear insights into rationale, contexts and/or processes related to why health systems pursue disinvestment and how they engage in and implement approaches to disinvestment.

🞎 **Yes**

🞎 **No**

1. **Conceptual mapping (Please check all boxes that apply)**

🞎 Agenda setting/prioritization for disinvestment

🞎 Policy development related to disinvestment

🞎 Implementation of disinvestment initiatives at the mezzo or macro level

🞎 Rationale for disinvestment

🞎 Political and health system context related to disinvestment

🞎 Processes for disinvestment
