# Supplementary material for: Processes, contexts, and rationale for disinvestment: a protocol for a critical interpretive synthesis
Source: Syst Rev. 2014 Dec 11;3:143. doi: 10.1186/2046-4053-3-143 (PMC4273322; doi:10.1186/2046-4053-3-143)
Supplement: Supplementary file 3 — Additional file 3: Data extraction framework. Approached to be used to extract data from included literature. (DOCX 26 KB) [file 13643_2014_309_MOESM3_ESM.docx]

**Additional File 3 – Data Extraction Framework**

1. **Data extractor:**
2. **RefID (or list source if not from database searches):**
3. **Title:**
4. **Authors:**
5. **Describe the focus of the document (using one phrase if possible – e.g.,** policy processes for disinvestment from existing, ineffective health care practices)
6. **Summary of key findings or insights from the document (1-2 paragraphs)**
7. **Document characteristics** (check all the apply)
8. Year of publication:
9. Years of data collection (if applicable)
10. Methods used/type of paper
    1. Primary research

🞎 Systematic review (needs to have explicit search and selection criteria)

🞎 RCT

🞎 Cross-sectional

🞎 Cohort study

🞎 Interrupted time series

🞎 Before-after study

🞎 Qualitative study

🞎 Case study

🞎 Mixed methods (select other methods as applicable)

🞎 Other (specify)

- 1. Non-research

🞎 Review (not systematic)

🞎 Theory/discussion/policy or position paper

🞎 Commentary/editorial

🞎 Website content (e.g., Choosing Wisely website)

- 1. Publication status

🞎 Peer-reviewed journal

🞎 Grey literature

1. Country or region focus
   1. 🞎 General/global focus
   2. 🞎 Specific

🞎 High-income country(ies)

- Number of countries:
- List specific countries:

🞎 Low- and middle-income country(ies)

- Number of countries:
- List specific countries:

1. Academic discipline (select all that apply)

🞎 Health services, systems and policy

🞎 Health technology assessment

🞎 Economics

🞎 Population and public health

🞎 Clinical and Epi

🞎 Political science

🞎 Organization/management/administration

🞎 Other (specify)

1. **Frameworks and questions for extracting key findings**

Check the dependent and independent variables that the paper addresses and provide a brief summary of the information in the paper as it relates to the data extraction questions associated with it.

| **Dependent variables** | **Independent variables** | **Data extraction question(s)** | **Brief summary of information related to the data extraction questions** |
| --- | --- | --- | --- |
| 🞎 **Government agendas** (i.e., why health systems pursue disinvestment) | 🞎 Problem | Explain whether and how the paper offers insights into the problem of disinvestment based on a:   - focusing event; - change in an indicator; or - feedback from the operation of a current program or policy. |  |
|  | 🞎 Policies/solutions | Explain whether and how the paper offers insights into policies/solutions for disinvestment based on:   - diffusion of ideas; - feedback from the operation of an existing policy or program; - communication/persuasion; - whether the policy viewed as technically feasible; - if the policy fits with the dominant values and current national mood; or - if it is acceptable in terms of current budget workability or likely political opposition or support. |  |
|  | 🞎 Politics | Explain whether and how the paper offers insights into the politics of disinvestment based on:   - swings in national mood; - change in the balance of organized forces; or - Events within government |  |
| 🞎 **Policy development** (i.e., how health systems engage in and implement approaches to disinvestment) | 🞎 Institutions | Explain whether and how the paper offers insights about disinvestment related to:   - government structures (e.g., federal versus unitary government); - policy legacies (e.g., key past policies such as the Canada Health Act that shape, facilitate and/or constrain future policy); or - policy networks (e.g., executive council-appointed committees that involve a small number of key stakeholders vs. several arms-length interest groups each vying for the attention of political elites but with no formalized networks in place). |  |
|  | 🞎 Interests | Explain whether and how the paper offers insights about disinvestment related to:   - types of interest groups that may be involved (e.g., societal interest groups, elected officials, civil servants or researchers); - the specific interests in disinvestment each group may have; and - the influence/power each group might be able to wield. |  |
|  | 🞎 Ideas | Explain whether and how the paper offers insights about disinvestment related to:   - knowledge/beliefs about ‘what is’ (e.g., research knowledge); and - views about ‘what ought to be’ (e.g., values). |  |
|  | 🞎 External events | Explain whether and how the paper offers insights about disinvestment related to external events such as recessions, court decisions, etc. |  |
| 🞎 **Health system context** (cross-cutting variables) | 🞎 Governance arrangements | Explain whether and how the paper offers insights related to:   - policy authority (i.e., who makes policy decisions, how, using what types of frameworks, and on what terms); - organizational authority (i.e., who makes organizational decisions, how, using what types of frameworks, and on what terms); - commercial authority (i.e., who makes commercial decisions, how, using what types of frameworks, and on what terms); - professional authority (i.e., who makes professional decisions, how, using what types of frameworks, and on what terms); or - consumer & stakeholder involvement (i.e., how stakeholders are involved and on what terms). |  |
|  | 🞎 Financial arrangements | Explain whether and how the paper offers insights about disinvestment related to:   - financing systems (i.e., mechanisms used to raise revenue for a particular health system); - funding organizations (i.e., mechanisms used to pay for/purchase services from healthcare organizations within a health system); - remunerating providers (i.e., mechanisms used to pay for/purchase services from, individual providers within a health system); - purchasing products and services (i.e., mechanisms used to pay for/purchase products and services); or - incentivizing consumers (i.e., financial or non-financial mechanisms to change specified behaviours of those who receive care). |  |
|  | 🞎 Delivery arrangements | Explain whether and how the paper offers insights about disinvestment related to:   - how care is designed to meet consumers’ needs (i.e., the approaches taken to ensure care is delivered in a way that is sensitive to the needs of consumers); - by whom care is provided (i.e., the way health human resources are organized and used in the health system); - where care is provided (i.e., how the physical elements of the health system are organized); or - with what supports is care provided (i.e., the supports used to assist those providing and receiving care). |  |
